# Effects of the *Campylobacter jejuni* CJIE1 prophage homologs on adherence and invasion in culture, patient symptoms, and source of infection

**DOI:** 10.1186/1471-2180-12-269

**Published:** 2012-11-20

**Authors:** Clifford G Clark, Christopher CR Grant, Frank Pollari, Barbara Marshall3, Jason Moses, Dobryan M Tracz, Matthew W Gilmour

**Affiliations:** 1 National Microbiology Laboratory, Public Health Agency of Canada, 1015 Arlington Street, Winnipeg, Manitoba, R3E 3R2, Canada; 2 Department of Medical Microbiology, Room 543 – 745 Bannatyne Avenue, University of Manitoba, Winnipeg, Manitoba, R3E 0J9, Canada; 3 Surveillance Division, Centre for Foodborne, Environmental, and Zoonotic Infectious Diseases, Public Health Agency of Canada, 255 Woodlawn Road West, Unit 120, Guelph, Ontario, N1H 8J1, Canada

**Keywords:** C. jejuni, Prophage, Adherence, Invasion, Patient symptoms, Source of infection

## Abstract

**Background:**

Prophages of enteric bacteria are frequently of key importance for the biology, virulence, or host adaptation of their host. Some *C. jejuni* isolates carry homologs of the CJIE1 (CMLP 1) prophage that carry cargo genes potentially involved in virulence. Possible role(s) of CJIE1 homologs in the biology and virulence of *C. jejuni* were therefore investigated by using *in vitro* cell culture assays and by assessing the association of *C. jejuni* isolates with and without these prophages with patients’ symptoms, with source, and with clonal lineages within the *C. jejuni* population.

**Results:**

Four *C. jejuni* isolates, three carrying the CJIE1-like prophage and one without, were tested in cell culture assays for adherence and invasion. Both adherence and invasion of *C. jejuni* to cells in culture were increased by the presence of the CJIE1-family prophage. Differences in motility and growth rate did not appear to be responsible. The CJIE1 prophage was present in 23% of isolates from human and non-human sources combined that were obtained through sentinel-site surveillance, and the distribution of CJIE1 in this population showed modest clonal associations. There was no correlation between the presence of the CJIE1 prophage in *C. jejuni* and patient symptoms, although there was some statistical support for lower rates of abdominal pain and fever when the prophage was present. Little evidence was found for a role of the prophage in host adaptation or host specificity.

**Conclusion:**

These biological effects suggest that the presence of the prophage may be a marker for differential virulence of some *C. jejuni* isolates. Ongoing research into the effects of the prophage on protein expression may provide additional insights into the roles the prophage may play in the biology of its host bacterium.

## Background

Bacteriophages have critically important roles in genome diversification and the evolution of virulence and host adaptation of enteric bacteria. Genes encoding Shiga toxins (Stx) 1 and 2 are found on lambdoid phages in Shiga-toxigenic *Escherichia coli*, while similar Gifsy and Fels phages encode a number of virulence factors in *Salmonella enterica* serovar Typhimurium. In addition to carrying genes encoding virulence factors, integrated prophage can affect gene expression of the host bacterium.

The recent demonstration of three distinct bacteriophages integrated into the genome of *Campylobacter jejuni* chicken isolate RM1221 suggested that such phages may be common and important for the biology of *C. jejuni*[1]. At least one of these three *C. jejuni* integrated elements (CJIEs) [2] was a Mu-like phage inducible with mitomycin C designated CJIE1 (or *Campylobacter* Mu-like phage 1, CMLP1). Elements similar to these CJIEs were found quite frequently when a large panel of isolates was tested using a DNA microarray, and CMLP1 appeared to integrate essentially randomly in the genome [2]. Results from Southern blotting using CMLP1 genes as probes also showed that this phage appeared to be capable of loss and insertion or re-insertion into different parts of the *C. jejuni* genome, producing changes in pulsed-field gel electrophoresis (PFGE) patterns [3], and induction of prophages was found to be responsible for extensive genomic rearrangements in bacteria subject to predation by lytic bacteriophages [4]. Partial sequencing of a panel of 12 homologs of CMLP1 suggested these prophages have a mosaic structure due to recombination but did not identify inserted genes [5]. Recent work has identified putative inserted genes after completely sequencing four of these prophages [6]. The translation product of one of these indels, ORF11, was a hypothetical protein with no described function and an extremely limited distribution outside the prophages characterized. Proteomics experiments verified that this protein was expressed when isolates were grown on normal laboratory medium and up-regulated in the presence of bile salts (unpublished results).

This work was undertaken to determine whether the prophages associated with a group of highly related *C. jejuni* isolates affected the biology or virulence of the bacteria. Isolates carrying the prophage demonstrated higher levels of adherence and invasion in cell culture assays than those without. The presence of the prophage did not appear to greatly affect the severity of patient symptoms, host specificity, or host adaptation.

## Results

### Strain characteristics

The set of isolates used consisted of three *C. jejuni* isolates (00–2425, 00–2538, 00–2544) that carried a prophage homologous with, and closely related to, CJIE1 from strain RM1221 [1,6]. One isolate, 00–2426, did not carry the prophage. A few putative differences in gene content were detected in these four isolates using comparative genomic hybridization. PCR for these genes was done to confirm absence or divergence (primers are found in Table 1), and isolates were considered positive for the gene if it was present in either microarray or PCR analysis. No differences in gene content were found after the results of these analyses were completed and the isolates were considered genetically indistinguishable except for the lack of the CJIE1-family prophage in 00–2426; this evidence was crucial for allowing the research to proceed further.

**Table 1:** **PCR primers and conditions used in this study to verify the presence of genes associated with *C. jejuni *strains NCTC 11168 and RM1221 in isolates 00–2425, 00–2426, 00–2538, and 00-2544**

**Locus**	**Primer**	**Primer sequence 5' - 3'**	**Product** **size (bp)**	**Annealing temperature (°C)**
cj0032	F	TTTAAAGGCCAAGATAGAA	512	48.3
	R	GCGTAAAGAAATAGCAAGTT		
cj0138	F	GAAGGCGGGGTAAATCT	151	46.1
	R	TTGCAAAATGTTCTATCTT		
cje0302	F	TCCTTTGATGCTTTCTAA	137	43.8
	R	GTCCTATACTAACTCCACTT		
cj0424	F	TTTGTAGTTTAATTGCGATGTTGT	346	50.1
	R	AGTAGTTTTCCCTTTGCTCTCA		
cj0589	F	ATGGGGCTTTATTAGTTATT	547	46.6
	R	TCGCTTGATCTTACACCT		
cj0628	F	ATCAAAACAATTCGGCAACTT	455	51.5
	R	ACTTCGATTCAATATACCAACACC		
cj0780	F	TGGCGTTAAAGCGGGTGATA	492	52.8
	R	CCTGGTTTTGGGTTGATAGTCTT		
cj1158	F	TTTAAACATATCATAAGCACCTTTTT	107	46.0
	R	GCTATTACTTCTCCCGTGATTTAT		
cj1202	F	ATCAAAAATCTTCATGCTATCTTA	434	48.8
	R	TTATCTGTTCCTGCATTTACCTTA		
cj1218	F	AATTCTTTCGACTTCTTCC	317	46.6
	R	ATTTTATCGGCACACTTGA		
cj1318/ cj1336	F	GGAGGAAATGGAAAAGTTGAA	477	48.2
	R	AAATTGAGTACGCAGAGGTTGT		
cj1333	F	TTTTGGGGAATTTGATAAGGA	460	44.6
	R	ACAGTTGTAGGTGGTAATA		
cj1463	F	AAAGCCTTAAAAGAACAAACCAA	174	48.8
	R	TGAAAAACCCATACCTCCACTTA		
cj1622	F	ACGCCTTACATGAGTTTAT	438	48.4
	R	TAGGGCAATCTTTTCTTATG		
cj1729	F	CCATCTGCCGTTACTACTACTTTT	441	52.2
	R	ACAGGCTGGAACACCGACTATTA		

The “cj” locus designations refer to genes present in NCTC 11168, while the “cje” locus designation refers to a gene in RM1221.

All four isolates appeared fully motile when grown in semi-solid agar at 37°C under microaerobic conditions. The mean diameter of swarming growth in mm was as follows: 00–2426 (n = 12), 30.3 ± 7.4; 00–2425 (n = 12) 31.0 ± 4.7; 00–2538 (n = 9), 33.4 ± 5.2; 00-2544 (n = 11), 32.5 ± 3.7. Analysis using the One Way ANOVA indicated that there was no significant statistical difference between strains (Normality test passed, P = 0.470; Equal Variance test passed, P = 0.192, Power of Performed test with alpha = 0.50 was 0.049, below the desired power of 0.800), though the low value for the latter measure indicates results should be interpreted cautiously.

**Figure 1:**
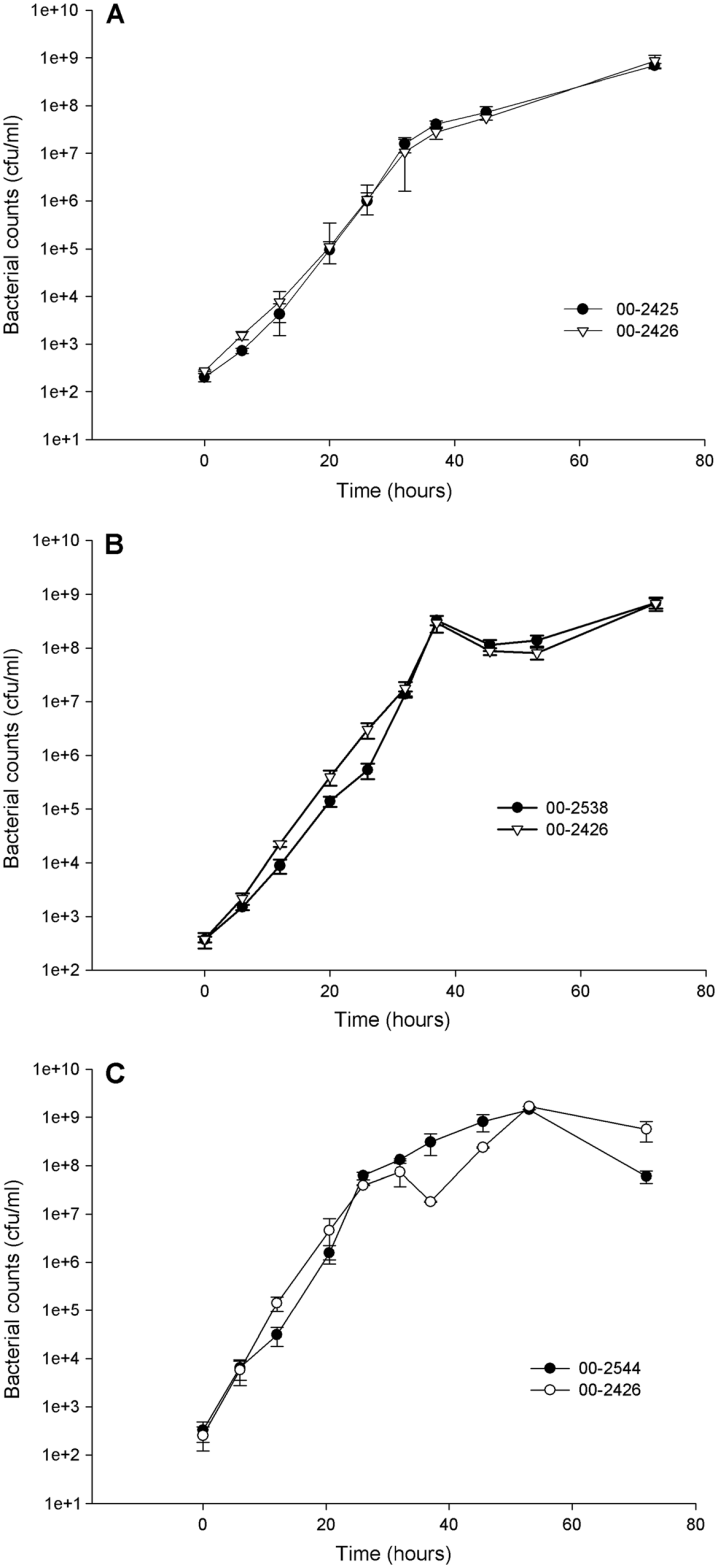
**Growth curves for *C. jejuni *isolates.A**. isolate 00-2425, with prophage vs. 00-2426, without prophage; **B**. isolate 00-2538, with prophage vs. 00-2426, without prophage; **C**. isolate 00-2544, with prophage vs. 00-2426, without prophage. Each set of paired growth curves was done during the same week from independent cultures as summarized in the Materials and Methods

Growth curves were done to determine whether the presence or absence of the CJIE1-family prophage affected growth of the organism. Each growth curve experiment compared one of the isolates carrying the CJIE1 prophage homolog with isolate 00–2426, which lacked the prophage (Figure 1). The growth curves shown are representative of the results from a number of growth curve experiments. There were subtle differences in growth in mid-log phase, with 00–2425, 00–2544, and 00–2538 all growing slightly faster (steeper slope of the line) than 00–2426. Though extremely subtle, this appeared to be consistent between experiments. Doubling times in mid-log phase were between 1.5 h and 2 h depending on the experiment.

### Adherence and invasion studies

**Figure 2:**
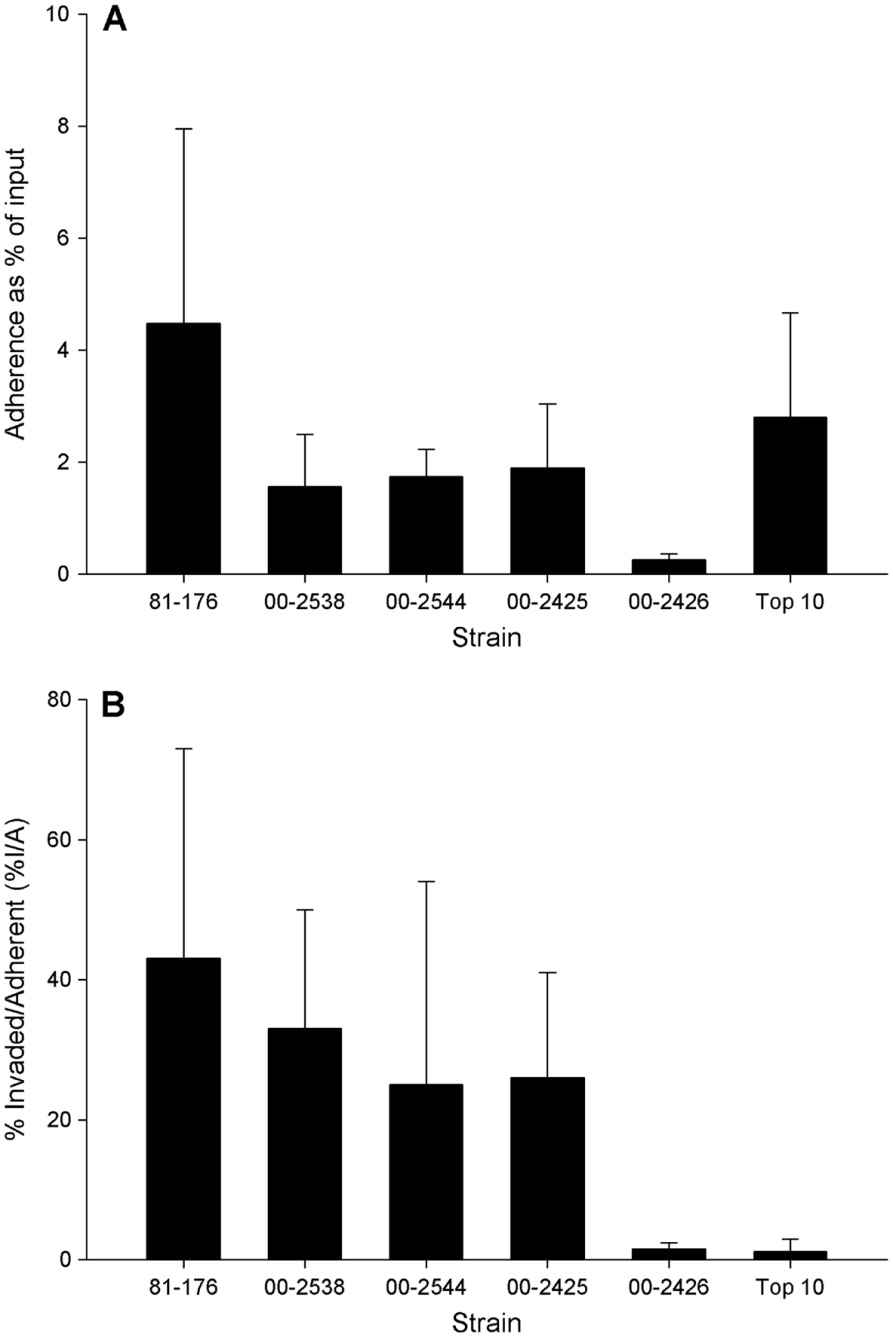
**Comparison of the adherence and invasion of *C. jejuni *test isolates and controls strains.A**. adherence as % of input, **B**. invasion expressed as the % invaded divided by the total bacteria adherent. Experimental replicates: 81-176, *n* = 10; 00-2538, *n* = 9; 00-2544, *n* = 5; 00-2425, *n* = 8; 00-2426, *n* = 6; *E. coli* Top 10 strain, *n* = 10

Isolates carrying the CJEI1 prophage homologs showed a moderate, but reproducible, difference in adherence and invasion (Figure 2A, Table 2). Control strain *C. jejuni* 81–176 was, on average, about 3-fold more adherent than the other *C. jejuni* isolates, and was 17-fold more adherent than isolate 00–2426 (unadjusted P = 0.000125). When all experimental replicates were assessed together the adherence levels of the three isolates carrying the prophage were approximately 6- to 7-fold that of the isolate without the prophage (Figure 2), though statistical significance was not reached in pairwise comparisons by One-way ANOVA with the Holm-Sidak Test. Despite this, experiments in which all four test strains were included were consistent in the trend to greater adherence among isolates carrying the prophage (Table 2).

**Table 2:** Comparison of three separate adherence and invasion experiments

**Isolate used**	**Input bacteria (cfu/ml)**	**Adherent bacteria (cfu/ml)**	**Adherent bacteria** **as % of input**	**Invaded bacteria (cfu/ml)**	**Invaded bacteria** **as % of input**	**% Invaded/adherent (%I/IA)**
**Experiment 1**						
81-176 (+ve control)	(5.2 ± 0.8) × 10^7^	(3.3 ± 1.0) × 10^6^	6.5	(6.3 ± 2.2) × 10^5^	1.22	18.8
00-2544 (+ prophage)	(3.7 ± 0.2) × 10^7^	(3.8 ± 1.2) × 10^5^	1.0	(2.7 ± 1.1) × 10^4^	0.07	7.0
00-2538 (+ prophage)	(3.7 ± 0.9) × 10^7^	(8.7 ± 0.1) × 10^5^	2.4	(2.0 ± 0.8) × 10^5^	0.54	23.0
00-2425 (+ prophage)	(4.1 ± 0.4) × 10^7^	(6.4 ± 0.8) × 10^5^	1.6	(2.0 ± 0.4) × 10^5^	0.48	31.9
00-2426 (− prophage)	(4.1 ± 0.1) × 10^7^	(1.2 ± 0.4) × 10^5^	0.3	(1.0 ± 0.7) × 10^3^	0.002	0.8
*E. coli* Top10 (invasion -ve)	(2.2 ± 0.1) × 10^7^	(5.2 ± 1.0) × 10^5^	2.3	(9.5 ± 0.2) × 10^3^	0.042	1.8
**Experiment 2**						
81-176 (+ve control)	(2.1 ± 0.5) × 10^7^	(3.5 ± 1.2) × 10^5^	1.6	(2.2 ± 0.3) × 10^5^	1.04	64.3
00-2544 (+ prophage)	(1.8 ± 0.8) × 10^7^	(3.5 ± 2.4) × 10^5^	1.9	(6.5 ± 1.5) × 10^4^	0.33	18.8
00-2538 (+ prophage)	(3.4 ± 0) × 10^7^	(3.9 ± 2.3) × 10^5^	1.1	(7.0 ± 0.9) × 10^4^	0.20	17.9
00-2425 (+ prophage)	(3.3 ± 0.6) × 10^7^	(5.6 ± 1.5) × 10^5^	1.7	(8.4 ± 3.5) × 10^4^	0.26	15.0
00-2426 (− prophage)	(3.4 ± 0.2) × 10^7^	(4.0 ± 2.1) × 10^4^	0.1	(8.7 ± 3.3) × 10^2^	0.003	2.2
*E. coli* Top10 (invasion -ve)	(1.8 ± 0.2) × 10^7^	(1.7 ± 0.9) × 10^5^	1.0	(9.6 ± 1.6) × 10^3^	0.055	5.7
**Experiment 3**						
81-176 (+ve control)	(3.4 ± 0.1) × 10^7^	(1.8 ± 0.3) × 10^6^	5.4	(1.0 ± 0.6) × 10^5^	0.30	5.6
00-2544 (+ prophage)	(2.3 ± 0.3) × 10^7^	(3.6 ± 1.3) × 10^5^	1.6	(4.1 ± 2.0) × 10^4^	0.18	11.4
00-2538 (+ prophage)	(3.8 ± 0.4) × 10^7^	(6.3 ± 2.8) × 10^5^	1.7	(1.3 ± 0.3) × 10^5^	0.33	20.2
00-2425 (+ prophage)	(4.3 ± 1.0) × 10^7^	(1.1 ± 0.2) × 10^6^	2.5	(2.5 ± 1.0) × 10^5^	0.58	22.8
00-2426 (− prophage)	(3.8 ± 1.6) × 10^7^	(1.6 ± 0.3) × 10^5^	0.4	(5.5 ± 6.0) × 10^2^	0.001	0.35
*E. coli* Top10 (invasion -ve)	(2.0 ± 0.01) × 10^7^	(2.1 ± 0.6) × 10^5^	1.1	(4.9 ± 2.2) × 10^3^	0.024	2.3

To assess invasion, differences in adherence are controlled for by calculating the % invaded/% adhered ratios (%I/%A). Invasion was associated, independently of adherence, with carriage of the CJIE1 prophage homolog (Figure 2B). Control strain 81–176 exhibited about 28-fold greater invasion than 00–2426 (unadjusted P = 0.0000149). Isolate 00–2538, which carries the prophage, was 21-fold more invasive than 00–2426, a statistically significant result in pairwise comparisons using the Holm-Sidak method (unadjusted P = 0.000769). Prophage-carrying isolates 00–2544 and 00–2425 were 16-fold and 17-fold more invasive than isolate 00–2426 lacking the prophage. These results were not statistically significant in pairwise comparisons in One-way ANOVA using the Holm-Sidak Test. *E. coli* Top 10 was used as a negative control for invasion; the levels of invasion of isolate 00–2426 and Top 10 were very similar throughout the experiments. Once again, the observation that isolate 00–2426 was much less invasive than the other *C. jejuni* strains was observed consistently in experiments in which all isolates were tested within a single experiment, on the same day (Table 2).

### Association of prophage with patient symptoms and source

Data on patient symptoms and the associated *C. jejuni* recovered from the patients was obtained through a collaboration between the National Microbiology Laboratory and the Centre for Foodborne, Environmental, and Zoonotic Infectious Diseases in Guelph, ON, which administers the C-EnterNet sentinel site surveillance program in the Region of Waterloo, ON [7]. This has allowed comparisons of the CJIE1 prophage genotype with patient symptoms. The PCR method developed for single-step detection of CJIE1 also assesses the presence or absence of an indel or moron carrying the unique coding sequence ORF11 [6]. Results are summarized in Table 3 and can be interpreted as in the following example. Of all 204 patients answering the question of whether they had abdominal pain, for instance, 169 answered “yes” and the remainder answered “no”. Among the 153 patients from whom *C. jejuni* without CJIE1 was isolated and who also answered the question on the questionnaire, 127 had abdominal pain and 26 did not. Similar interpretation can be applied throughout the table. As a whole these analyses suggested that the presence of ORF11 may be responsible for higher rates of bloody diarrhea and hospitalization and lower rates of headache, while the presence of the CJIE1 prophage was associated with lower rates of vomiting and longer duration of illness. None of these differences were statistically significant. Differences in the rates of abdominal pain and fever were significant, with higher rates observed from isolates lacking CJIE1 (P = 0.037 and P < 0.001, respectively). In both cases the difference in rates remained significant when rates of each symptom were compared pairwise between isolates without CJIE1 and those with CJIE1 alone (abdominal pain P < 0.025, fever P < 0.001; Fisher Exact Test) but were not significant when isolates with CJIE1 + ORF11 were compared with isolates carrying either CJIE1 only or no CJIE1 at all.

**Table 3:** Association of the CJIE1 prophage and the CJIE1 prophage carrying ORF 11 with patient symptoms

**Symptoms**	**Patients with symptoms (%) versus total**	**Association of *C. jejuni *strain characteristics with symptoms: number associated with patient and symptom vs total (%)**		
		**No CJIE1 (%)**	**CJIE1 only (%)**	**CJIE1 + ORF 11**
Diarrhea	214/218 (98.2)	158/162 (97.5)	16/16 (100)	15/15 (100)
Abdominal pain	169/204 (83.0)	127/153 (83.0)	9/16 (56.3)	12/15 (80.0)
Fever	134/219 (61.2)	107/146 (73.3)	4/16 (25.0)	6/14 (42.9)
Malaise	127/199 (63.8)	95/145 (65.5)	9/16 (56.3)	9/14 (64.3)
Nausea	113/205 (57.5)	87/151 (57.6)	8/16 (50.0)	9/14 (64.3)
Headache	91/201 (45.3)	70/142 (49.3)	7/16 (43.8)	4/11 (36.4)
Bloody diarrhea	49/145 (33.7)	33/99 (33.3)	4/15 (26.7)	8/14 (57.1)
Vomiting	73/214 (34.1)	56/157 (35.7)	3/16 (18.8)	5/14 (35.7)
Duration > 10 days	33/137 (24.1)	35/102 (34.3)	2/10 (20.0)	3/9 (33.3)
Hospitalization	15/142 (10.6)	10/125 (6.6)	1/13 (7.7)	2/13 (15.4)

Note that there were different response rates for different questions, resulting in different denominators. “Patients with symptoms” refers to the number of patients having the particular symptom compared with the total number of patients answering the question yes or no on the questionnaire. This column provides data on the overall frequency of symptoms. Isolates for further analysis were not available for all patients answering the comprehensive questionnaire. Data in the section “Association of *C. jejuni* strain characteristics with symptoms…” contains symptom information from patients from whom isolates were obtained and were typed. The frequencies with which each symptom was associated with the presence of absence of the CJIE1 prophage and also the presence within the CJIE prophage of ORF11 have been compared to determine whether either CJIE1 alone or CJIE1 with ORF11 have any significant effect on patient symptoms compared with absence of the prophage.

C-EnterNet also recovers bacteria from food, animals, and environmental sources within the sentinel site. These isolates were used to assess whether there was any association between the presence of the CJIE1 prophage or the CJIE1 prophage + ORF11 and recovery of *Campylobacter* spp. from particular sources. The data summarized in Table 4 indicate that there was a much higher percentage of *C. jejuni* isolates without the CJIE1 prophage from water than from chicken breast, humans, and pigs (P = 0.003 for comparison of water with retail chicken breast, P = <0.001 for other comparisons). A higher number of *C. jejuni* without the CJIE1 prophage was also found in isolates from bovine manure (P = 0.027) compared with isolates from retail chicken breast. The carriage of CJIE1 and CJIE1 + ORF11 was significantly higher in *C. coli* in isolates from chicken than those from humans (P = 0.003). Other differences were noted but not tested for statistical significance because of the small numbers involved (Table 4).

**Table 4:** Distribution of CJIE1 prophage and ORF 11 according to source and bacterial species

**Source of isolate**	**Species**	**Total**	**No prophage (%)**	**Prophage alone (%)**	**Prophage + ORF 11 (%)**
chicken breast	*C. jejuni*	113	82 (73)	24 (21)	7 (6)
	*C. coli*	14	7 (50)	6 (43)	1 (7)
river water	*C. jejuni*	14	12 (86)	1 (7)	1 (7)
human	*C. jejuni*	244	190 (78)	27 (11)	27 (11)
	*C. coli*	25	17 (68)	8 (32)	0
bovine manure	*C. jejuni*	33	29 (88)	3 (9)	1 (3)
swine manure	*C. jejuni*	4	1 (25)	3 (75)	0
	*C. coli*	21	21 (100)	0	0
other non-human	*C. jejuni*	11	9 (82)	1 (9)	1 (9)
	*C. coli*	6	6 (100)	0	0
Total		496	381 (77)	74 (15)	41 (8)

There appeared to be a somewhat stronger association of ORF11 with multi-locus sequence types (ST) 21, 48, 460, 508, and 982 (Table 5). Similarly, CJIE1 alone was found more frequently in STs 42 and 50. Associations of CJIE1 alone with other STs were less certain due to the small number of isolates detected for each ST. All isolates belonging to STs 137, 429, and 939 carried CJIE1, but only 2–3 isolates of each ST were available. Similarly, ORF 11 was found with higher frequency in *flaA* SVR types 34, 36, 46, 49, and 172 (Table 6).

**Table 5:** Frequency of carriage of CJIE1 prophage and ORF11 within the predominant multi-locus sequence types (ST)

**ST**	**Total in each ST**	**No CJIE1** **no Orf 11 (%)**	**With CJIE1** **alone (%)**	**With CJIE1** **+ ORF11 (%)**
8	10	9/10 (90)	0	1/10 (10)
21	25	16/25 (64)	4/25 (16)	5/25 (20)
42	15	8/15 (53)	7**/**15 (47)	0
45	91	84/91 (92)	5/91 (6)	2/91 (2)
48	11	8/11 (73)	0	3/11 (27)
50	9	7/9 (78)	2/9 (22)	0
52	7	7/7 (100)	0	0
61	7	7/7 (100)	0	0
137	2	0	2/2 (100)	0
267	8	7/8 (88)	1/8 (12)	0
353	8	7/8 (88)	1/8 (12)	0
429	3	0	3/3 (100)	0
459	15	14/15 (93)	0	1/93 (7)
460	9	7/9 (78)	1/9 (11)	1/9 (11)
508	7	6/7 (86)	0	1/7 (14)
806	8	7/8 (88)	1/8 (12)	0
922	13	13/13 (100)	0	0
929	13	13/13 (100)	0	0
939	2	0	2/2 (100)	0
982	38	27/38 (71)	5/38 (13)	6/38 (16)
1068	14	12/14 (86)	2/14 (14)	0
Total	315	259/315 (82)	36/315 (11)	20/315 (6)

**Table 6:** **Frequency of carriage of CJIE1 prophage and ORF11 within the predominant *flaA *SVR types**

***flaA *SVR**	**Total in each type**	**No CJIE1 no Orf 11 (%)**	**With CJIE1 alone (%)**	**With CJIE1 + ORF11 (%)**
2	7	7/7 (100)	0	0
5	6	5/6 (83)	0	1/6 (17)
8	13	10/13 (77)	3/13 (23)	0
9	8	7/8 (88)	1/8 (12)	0
11	6	5/6 (83)	1/6 (17)	0
13	12	10/12 (83)	2/12 (17)	0
14	28	26/28 (93)	1/28 (3.5)	1/28 (3.5)
16	23	21/23 (91)	2/23 (9)	0
18	16	14/16 (88)	2/16 (12)	0
21	59	55/59 (93)	2/59 (3.5)	2/59 (3.5)
22	13	12/13 (92)	1/13 (8)	0
34	18	12/18 (67)	4/18 (22)	2/18 (11)
36	24	14/24 (58)	2/24 (8)	8/24 (33)
41	10	9/10 (90)	1/10 (10)	0
42	10	10/10 (100)	0	0
45	5	5/5 (100)	0	0
46	14	10/14 (72)	2/14 (14)	2/14 (14)
49	45	34/45 (76)	5/45 (11)	6/45 (13)
57	8	8/8 (100)	0	0
70	8	6/8 (75)	2/8 (25)	0
122	9	9/9 (100)	0	0
172	12	8/12 (66)	2/12 (17)	2/12 (17)
239	13	10/13 (77)	2/13 (15)	1/13 (8)
245	3	0	3/3 (100)	0
359	27	22/27 (81)	5/27 (19)	0
840	8	8/8 (100)	0	0

## Discussion

CJIE1-positive isolates were around 6 to 7-fold more adherent and 16 to 21-fold more invasive for INT-407 cells in culture than the isolate that did not carry this prophage. These values are close to those found when other genes have been examined; a similar 7-fold increase in adherence to INT-407 cells was found with a cj1461 (methyltransferase) mutant versus the wild-type strain [8]. Mutants in *waaF* showed a 14-fold reduction in invasion of INT407 cells compared with the wild type strain [9]. Disruption mutants of adhesin-encoding genes *cadF* and *flpA* exhibited a 72% and 62% reduction in adherence, respectively [10]. Insertion mutagenesis of cj0588 encoding the TlyA product caused a significant reduction in adherence to Caco-2 cells in culture of *C. jejuni* strains 81–176 (decreased to 59% compared with wild type) and 81116 (reduced to 48% compared with wild type) [11]. Results from our assays were quite similar to these studies, showing a 0.5 to 1.0 log reduction in adherence of the isolate without the CJIE1-family prophage (Table 2).

The presence of the prophage therefore makes a substantial contribution to the adherence of the lysogenized bacterium. Though the trend to much higher adherence by isolates carrying the prophage was clear in all experiments, the differences in the adherence of isolates with and without the prophage did not reach statistical significance. This was likely partly due to the inter-experimental variability in the adherence and invasion assays, which has been noted before [12] and appears to be a characteristic of the assay. Differences in adherence *in vivo* can be very significant even when cell culture assays demonstrate no difference between strains [13]. It is critically important that the role of the prophage be assessed in a relevant animal model and with functional mutagenesis studies.

Invasion of Caco-2 cells was reduced in *tlyA* mutants to 56% and 31% of wild-type in *C. jejuni* strains 81–176 and 81116, respectively [11]. The 16- to 21-fold difference in invasion detected in the isolates with and without the CJIE1-family prophage was similar to this but much less than the 50-fold reduction in invasion of INT-407 cells resulting from an insertion mutation of cj1461 [8]. However, the cj1461 mutant also resulted in a motility defect, which is known to have profound effects on invasion [14,15]. In contrast, no gross alterations in motility were seen in *C. jejuni* isolates with and without the prophage in the present study. The relative numbers of invaded bacteria expressed as a percentage of those adherent at 30 min post-inoculation was higher than seen by Christensen *et al*. [16]. However, the differences between adherence and invasion of bacteria with and without the CJIE1-family prophage were consistent in all experiments, suggesting that whatever technical differences resulted in the higher %I/A values were also consistent. The measurable differences in adherence and invasion associated with prophage carriage found in this study appear to be substantiated. Of note, both strain 81–176 and 00–2538 had differences in adherence that were statistically significant when compared with isolate 00–2426. Furthermore, the levels of adherence and invasion expressed as percentage of input or inoculum counts was very similar to that found in other studies [17].

DNA sequencing of the CJIE1-1 prophage from isolate 00–2425 [6] has demonstrated the presence of a few genes associated with the prophage that are likely not important for prophage structure, life cycle, or replication, ie. that appear to be cargo genes, in addition to a number of hypothetical proteins. Among the putative cargo genes are: the CJE0220 homolog, a DAM methylase; ORF3, a KAP family P loop domain protein; a CJE0256 homolog, *dns*, an extracellular DNase; ORFs 10 and 11 inserted in the early region of the prophage with no homology to any protein of known function within GenBank. We speculate that the effects of the CJIE1-1 prophage on cells in culture are mediated either by a novel effector or by a regulator of virulence genes or even general metabolism within the *C. jejuni* bacterial cell. Differences in protein expression between isolates with and without CJIE1 in iTRAQ experiments support this hypothesis (unpublished data).

No consistent or statistically significant differences in motility were found when comparing isolates with and without the prophage. The differences in adherence and invasion were therefore not directly the result of differences in motility, and were also not likely to be due to differences in gene content, other than the previously noted prophage genes, or growth rate.

The four isolates used were all obtained at the same time and in the same place during an outbreak of disease. They were the same subtype and had indistinguishable gene content as measured by comparative genomic hybridization DNA microarray analysis except for the fact that isolate 00–2426 lacked the CJIE1-family prophage. Though a consistent difference in growth rate was seen during mid-logarithmic phase between the isolate lacking the prophage and the three isolates carrying the prophage, this difference was extremely subtle. It does not seem likely that this degree of difference could be responsible for the differences seen in adherence and invasion.

It must be noted that the combination of microarray data and calculation of genome sizes does not prove absolutely that the four isolates have identical DNA sequences other than the presence or absence of CJIE1. Because the microarray had probes for genes from only two strains it is possible that other genes or DNA segments could be present. However, calculation of genome sizes from PFGE fragments sizes was done previously with a reasonable degree of accuracy, and the resulting data indicate that genomes of the isolates 00–2425 and 00–2544 carrying CJIE1 differed from 00–2426, which lacked CJIE1, by 39 kb [3]. This constrains the variability that would be expected for the four genomes mainly to the presence or absence of the prophage and to DNA sequence changes arising from horizontal gene transfer. The use of three different, closely related isolates carrying the CJIE1 prophage at different places in the genome (as assessed through differences in PFGE patterns) should minimize the chances that differences arising from proximity of the prophage to specific genes, from point mutations, and from horizontal gene transfer. Whole genome sequencing of these isolates is planned for the near future and should provide unambiguous data regarding gene content and prophage location.

An unexpected observation unrelated to the investigation into prophages came from conducting growth curve experiments with *C. jejuni* for the first time. Very similar OD_600_ values were obtained for all four test strains after 48 h (early stationary phase) growth in initial experiments suggesting that, if differences existed between isolates, they were both quite subtle and quite growth phase-specific. Note that these subtle effects were visualized as occurring in mid-log phase (around 5 × 10^5^ cfu/ml) as measured by plating growing cultures, and would likely not have been observed if growth were measured using spectrophotometry, as growth was not detectable at OD_600_ until cell density was between 5 × 10^7^ to 1 × 10^8^ cfu/ml (data not shown).

Molecular typing data and information about patient symptoms were available for a relatively large number of human and non-human isolates obtained through the C-EnterNet sentinel site surveillance system. Though there appeared to be some association of ORF11 with bloody diarrhea and hospitalization, this did not attain statistical significance. A further, somewhat puzzling, observation was that the presence in *C. jejuni* of CJIE1 in the absence of ORF11 appeared to reduce the frequency of some symptoms (Table 3). This was statistically significant for abdominal pain and fever, though caution should be used in interpretation of the statistical analysis because only a relatively small number of isolates fit into this category. It should be noted that not all patients for which isolates were available filled out questionnaires, and isolates were not available for all patients who filled out questionnaires. It would be of interest to add to the observations in this study over time and determine whether any of the apparent trends are supported by further data.

Carriage of both the prophage and of ORF11 was less frequent in most *C. jejuni* isolates from water, suggesting these elements do not have adaptive value for the organism in this environment. Further research is required to verify this observation and to determine whether this is associated with the biology of the organism or purely stochastic in nature. Differences in the proportion of isolates with and without the CJIE1 prophage between *C*. *jejuni* isolates from chicken, human, and bovine sources were either slightly statistically significant (chicken and bovine, P = 0.027) or not significant (chicken and human, human and bovine). There is no strong evidence that the prophage or ORF11 play a role in host adaptation or host specificity.

A striking difference in the frequency of carriage of both CJIE1 alone and of CJIE1 + ORF11 in both STs and in *flaA* SVR types suggests that the carriage of these elements may be specific to certain *Campylobacter* lineages, groups, or clones. Prophage CJIE1 + ORF11 was found at higher frequency in ST 8, 21, 48, and 982. STs 21 and 982 differ only by a single allele and ST 8 is included with ST 21 in clonal complex 21, while ST 48 differs at three alleles from ST 21 and four alleles from ST 982. Similarly, CJIE1 alone is found at higher frequency in ST 21, 42, 50, and 982, and a few other STs, while it is found in much lower frequency in ST 45 and several additional STs (Table 5). One possibility is that the carriage and transmission of the CJIE1 prophage may be strongly associated with a specific animal host or environmental niche. MLST types exhibit a host-specific signature of alleles acquired through homologous recombination during carriage and adaptation of *Campylobacter* within the host species [18]. Studies in Finland indicate that the ST-45 clonal complex is significantly associated with chicken isolates, while the ST-21 and ST-48 clonal complexes are significantly associated with human isolates [19]. Clonal complexes ST-21 and ST-42 are also among the lineages that predominate among *C. jejuni* isolates from cattle [20]. Together this information might suggest that the CJIE1 prophage, like the host-specific MLST alleles, may be circulating in a subset of *C. jejuni* more closely associated with humans and cattle than with chickens. This finding supports the conclusions of Pittenger *et al*. [21], who determined that *C. jejuni* RM1221 variable genes – most of them of prophage origin – were more widely distributed in isolates from cattle and humans than from other sources. However, for CJIE1 it was apparent from the results presented in Table 4 that the prophage was present in a greater proportion of *C. jejuni* from chickens and swine manure than any other sources, though the number of isolates obtained from swine manure do not allow much confidence in that result. A great deal more research into the association of prophages and cargo genes carried by prophage elements is warranted.

## Conclusions

The presence of CJIE1 prophages affected both adherence and invasion of the lysogenized bacterium; these effects on adherence and invasion were not due to differences in motility or growth. They also did not appear to result from minor differences in the gene content of the isolates as evaluated by microarray analysis. It is therefore most likely that the prophage, or some gene or genes within the prophage such as ORF11, was responsible for the increased levels of both adherence and invasion. There was no strong evidence that the prophage or ORF11 play a role in host adaptation, host specificity, or human pathogenicity. Research into the effects of the prophage on protein expression is expected to provide additional insights into the roles the prophage may play in the biology of its host bacterium.

## Methods

### Isolate characterization

Isolates were originally obtained during the large waterborne outbreak of *C. jejuni* and *E. coli* O157:H7 in Walkerton, Ontario in 2000. Strain typing was done previously [22]. All four human clinical isolates were epidemiologically related as part of a large water-borne outbreak of *Campylobacter* in Ontario, Canada, in the year 2000 [22,23]. The isolates were also very closely related by phenotypic and genotypic typing tests (Table 7). Other than PFGE restriction profile, which we have previously shown resulted from movement of the prophage in the chromosomes [3], the only difference was that isolate 00–2544 was PT35 rather than PT33.

**Table 7:** **Characteristics of clinical *C. jejuni *isolates used for adherence and invasion assays (from Clark *et al*. [**[22]**,**[23]**])**

**Isolate**	**Bio type**	**ST**	***flaA *SVR type**	***fla*-RFLP**	**HS serotype**	**HL serotype**	**Phage type**	**PFGE *Sma *I**	**PFGE *Kpn *I**
00-2425	II	21	36	1	O:2	125	33	2	2
00-2426	II	21	36	1	O:2	125	33	1	1
00-2538	II	21	36	1	O:2	125	33	11	1
00-2544	II	21	36	1	O:2	125	35	4	1

ST, sequence type according to the Oxford MLST scheme; *flaA* SVR, sequence of the flagellin short variable region DNA sequence according to the Oxford scheme; *fla-*RFLP, restriction fragment-length polymorphism of the amplified complete *flaA* locus; HS, heat-stable or Penner serotype; HL, heat-labile or Lior serotype.

Isolates 00–2425, 00–2538, and 00–2544 all carried a prophage homologous to CMLP1 (CJIE1) of strain RM1221. Isolate 00–2426 lacked this prophage. The motility of each isolate was assessed by applying 10 μl of growth from Brucella broth adjusted to OD_600_ = 0.1 onto semi-solid agar (Oxoid Mueller-Hinton broth + 0.4% Select Agar). Zones of motility were measured after growth for 48 h at 37°C under microaerobic conditions.

### Growth curves

Bacteria grown on Oxoid Mueller Hinton Agar + 10% sheep erythrocytes were used to inoculate 50 ml BBL^TM^ Brucella Broth Albimi (VWR Canada, Mississauga, ON, Canada). After overnight growth, each suspension was diluted to an OD_600_ of approximately 0.18 to 0.2 (approximately 2 × 10^8^ cfu/ml). This suspension was diluted by 10^-4^ to a concentration of approximately 2 × 10^4^ cfu/ml and 0.5 ml of the resulting suspension was inoculated into 50 ml Brucella Broth Albimi to give approximately 200–500 cfu/ml. Growth proceeded for four days at 37°C under microaerobic conditions (5% O_2_, 10% CO_2_, 85% N_2_). At intervals aliquots were taken, diluted appropriately, and plated in duplicate onto Mueller-Hinton agar plates for determining viable cell counts. All plates with 20 – 300 colonies were counted, so that between 2 and 4 values were available for calculating the mean plus standard deviation of the cell concentration. Inoculated plates were incubated in microaerobic conditions at 42°C for 36 – 48 h, or at 37°C for 3 days, and colonies were counted. Data were plotted in Sigma Plot 10.0.1 (Systat Software Inc, San Jose, CA). Eight growth curve experiments produced usable data, and the three chosen for publication were representative.

### Microarray analysis for gene content of isolates

*C. jejuni* NCTC 11168 ORF amplicon arrays were provided by Dr. E. Taboada. This version of the array also included targets representing unique ORFs from *C. jejuni* RM1221.

Comparative genomic hybridization microarray analysis was performed according to previously described methods [24,25]. NCTC 11168 genomic DNA was included as the reference probe in all experiments. Genomic DNA was nebulized to produce fragments of approximately 1 to 5 kb. Fragmented DNA (5 μg) from each strain was labeled with either cyanine 3 (Cy3) or cyanine 5 (Cy5) fluorescent dye by direct chemical coupling using the Mirus Label-It Kit (Mirus Corp. Madison, Wis.) according to the manufacturer’s instructions. Unincorporated dye was removed by sequential passage of the labeled DNA through Mirus columns followed by columns included in the QiaQuick PCR Purification kit (Qiagen, Mississauga, ON, Canada).

Equal amounts (0.8 – 1.0 μg) of labeled genomic DNA from each strain were mixed, lyophilized, and suspended in hybridization buffer (90% DIG Easy Hyb [Roche, Laval, QC, Canada], 5% tRNA [Sigma, Oakville, ON, Canada], and 5% salmon sperm DNA [Invitrogen Canada Inc, Burlington, ON, Canada]). After incubation at 65°C for 5 min, probes were cooled to room temperature, added to microarray slides (75 μl probe volume) under Lifter Slip coverslips (Erie Scientific), and hybridized overnight at 37°C in hybridization chambers containing DIG buffer to provide humidity. After hybridization the microarrays were washed twice for 5 min each with 1 × SSC, 0.1% SDS, twice for 5 min each with 0.5 × SSC, and once for 1 min with 0.1 × SSC. At least two technical replicates and dye swap experiments were done for each test strain to allow appropriate data analysis.

Microarray slides were scanned in an Agilent scanner (Agilent Technologies, Mississauga, ON, Canada). Signal data were extracted with ArrayPro Analyzer version 4.5.1.48 (Media Cybernetics Inc., Silver Spring, MD) and compiled in Microsoft Excel spreadsheets. Normalization of data, as well as removal of batch effects due to technical and dye intensity variation, was performed with Partek-Pro™ statistical analylsis software (Partek Inc., St. Louis, MO). Log_2_ ratios of the data were obtained [24,25] and analysis of the overall relatedness of the genomes and identification of absent or divergent loci was done using GeneMaths software (Applied Maths, Austin, Tx).

### Description of PCR rationale, primers, and reaction conditions

PCR for verifying absence or divergence of loci was done using the primer sets summarized in Table 1 with reagents from FastStart Taq DNA Polymerase kits (Roche Diagnostics, Laval, QC, Canada) according to the instructions of the manufacturer. The final MgCl_2_ concentration used was 2.0 mM and between 20 – 250 ng of extracted DNA, obtained using a Gentra Systems PUREGENE DNA isolation kit (Qiagen), was used for amplification. Cycle parameters were: initial denaturation at 92°C, 5 min; 35 cycles of denaturation at 92°C for 30s, annealing for 1 min, and extension for 1 min at 72°C; 7 min final extension; storage at 4°C. Amplification products were visualized by agarose gel electrophoresis and ethidium bromide staining.

One gene pair, cj1318 and cj1336, had extensive overlapping regions of DNA sequence identity. The primers obtained could not differentiate the two genes; for the purposes of our discussion, positive results were taken to mean that both loci were present, though this has not been unambiguously demonstrated.

PCR was undertaken to detect the CJIE1 prophage and ORF11 from CJIE1. The PCR reaction primers and conditions have been described previously [6]. An amplification product of approximately 750 bp signified the presence of the CJIE1 prophage while a larger amplification product of approximately 1700 bp indicated the presence of the ORF11 indel. A total of 496 *Campylobacter* spp. isolates were tested using this PCR method.

### Adherence and invasion assays

Assays were done according to the methods of Malik-Kale *et al*. [26], except that wells were seeded with 2 × 10^7^ INT-407 cells the day before the assay to give a newly confluent monolayer at the time the assay began. Two strategies were used to perform the adherence and invasion assays. In the first series of experiments only two *C. jejuni* test isolates were assessed in each experiment along with the *C. jejuni* 81–176 and *E. coli* Top 10 control strains. This was done in order to manage the timing of steps and reduce the possibility of technical errors. Almost all of these experiments were done by a single technologist and the INT-407 cells used were between passages 65 – 120. Furthermore, a gentamicin concentration of 750 μg/ml was used to kill extracellular bacteria.

A second series of experiments was done to compare the adherence and invasion of all isolates and controls in a single experiment. Fresh INT-407 cells were obtained and used between passages 5 – 20. For these later experiments, the concentration of gentamicin was reduced to 500 μg/ml based on testing of the strains used. There were no obvious differences in results using either concentration of antibiotic. Results from all assays were used to create Figure 2 and perform the statistical analyses. Similarly, results from the second series of experiments were summarized in Table 2 to show the variability between experiments and common trends when comparing isolates carrying the CJIE1 prophages versus the isolate without the prophage.

Values for percent adherence (%A) and percent internalization divided by adherence (%I/A) were described previously [26]. The value for percent adherent was obtained from by dividing the values obtained for adherent bacteria (cfu/ml) by the values obtained for input bacteria (cfu/ml) and multiplying by 100. Similarly, the percentage invaded was obtained by dividing the values obtained for invaded bacteria (cfu/ml) by the values obtained for adherent bacteria (cfu/ml) and multiplying by 100. The adherence assay was done after incubating bacteria with INT-407 cells for 30 min, after which adherence is assumed to be close to maximal, and the invasion assay was begun after 3 h of incubation of bacteria with INT-407 cells [26].

It must be noted that INT-407 cells have been found to contain contaminating HeLa markers. However, they have been used extensively for testing the adherence and invasion of *Campylobacter jejuni*[8,10,12] and have been found useful in that respect. Use of these cells should provide acceptable information as long as there is no attempt to make inferences regarding *in vivo* situations.

### Sentinel site surveillance

C-EnterNet sentinel site surveillance in the Region of Waterloo, Ontario (human population of approximately 500,000) has been described previously [7], http://www.phac-aspc.gc.ca/c-enternet/index-eng.php. Isolates from both human and non-human (retail meats, on-farm manure, and surface water) sources from the sentinel site were characterized as part of the previous study. For each human case reported to the health unit a public health inspector contacted the patient to complete a comprehensive standardized questionnaire. Answers to the symptomology questions were collated and linked to the patient’s *Campylobacte*r isolate information.

### Statistical analysis

Statistical analysis for cell culture adhesion and invasion assays was done by using the One-way Analysis of Variance (ANOVA) performed using the Sigma Stat functions within the SigmaStat 3.5 software (Systat Software Inc.). The significance of each pairwise comparison was evaluated using the Holm-Sidak Test. The number of observations used for each factor is given in the legend to Figure 2. Swarming assay (motility) results were also assessed statistically by using the One Way ANOVA within SigmaStat 3.5 software.

The association of the presence of the CJIE1 prophage and the prophage + ORF11 with patient symptoms was analyzed using the Chi-Square analysis of contingency or the Fisher Exact Test within Sigma Stat 3.5 software.

## Competing interests

The authors declare no competing financial interests.

## Authors' contributions

Conceived and designed the work: CGC, MWG. Performed the laboratory experiments: CGC, CCRG, JM, DMT. Performed the analysis, including statistical analysis: CGC. Wrote the manuscript: CGC. Collected, collated, and provided patient and source data associated with the sentinel site: FP, BM. All authors read and approved the final manuscript.
